# Spatio-Temporal Variations and Influencing Factors of Country-Level Carbon Emissions for Northeast China Based on VIIRS Nighttime Lighting Data

**DOI:** 10.3390/ijerph20010829

**Published:** 2023-01-01

**Authors:** Gang Xu, Tianyi Zeng, Hong Jin, Cong Xu, Ziqi Zhang

**Affiliations:** 1 School of Architecture, Harbin Institute of Technology; Key Laboratory of Cold Region Urban and Rural Human Settlement Environment Science and Technology, Ministry of Industry and Information Technology, Harbin 150006, China; 2 School of Art and Design, Heilongjiang Institute of Technology, Harbin 150050, China; 3 Harbin Institute of Technology, Harbin 150006, China

**Keywords:** nighttime light data, low-carbon planning, county-level carbon emissions, Northeast China

## Abstract

This paper constructs a county-level carbon emission inversion model in Northeast China. We first fit the nighttime light data of the Visible Infrared Imaging Radiometer Suite (VIIRS) with local energy consumption statistics and carbon emissions data. We analyze the temporal and spatial characteristics of county-level energy-related carbon emissions in Northeast China from 2012 to 2020. At the same time, we use the geographic detector method to analyze the impact of various socio-economic factors on county carbon emissions under the single effect and interaction. The main results are as follows: (1) The county-level carbon emission model in Northeast China is relatively more accurate. The regression coefficient is 0.1217 and the determination coefficient R^2^ of the regression equation is 0.7722. More than 80% of the provinces have an error of less than 25%, meeting the estimation accuracy requirements. (2) From 2012 to 2020, the carbon emissions of county-level towns in Northeast China showed a trend of increasing first and then decreasing from 461.1159 million tons in 2012 to 405.752 million tons in 2020. It reached a peak of 486.325 million tons in 2014. (3) The regions with higher carbon emission growth rates are concentrated in the northern and coastal areas of Northeast China. The areas with low carbon emission growth rates are mainly distributed in some underdeveloped areas in the south and north in Northeast China. (4) Under the effect of the single factor urbanization rate, the added values of the secondary industry and public finance income have higher explanatory power to regional emissions. These factors promote the increase of county carbon emissions. When fiscal revenue and expenditure and the added value of the secondary industry and per capita GDP interact with the urbanization rate, respectively, the explanatory power of these factors on regional carbon emissions will be enhanced and the promotion of carbon emissions will be strengthened. The research results are helpful for exploring the changing rules and influencing factors of county carbon emissions in Northeast China and for providing data support for low-carbon development and decision making in Northeast China.

## 1. Introduction

To actively respond to the global climate change issue, our government pledged at the Paris Climate Conference to reduce carbon emission intensity by 60~65% by 2030 compared with 2005 [1,2]. Carbon emission reduction targets and limits must be distributed among provincial, municipal, and county levels; thus, an investigation of the spatial and temporal distribution patterns of carbon emissions in counties can provide a basis for more accurate emission reduction policies. However, the unique natural resource and historical development conditions in Northeast China (the three provincial administrative regions in Northeast China; the three northeastern provinces are divided into Liaoning Province, Jilin Province, and Heilongjiang Province) have formed many unique town-system structures built around the farming, forestry, coal, and oil systems [3]. The spatial pattern of these towns could be more manageable, with many problems such as small scale and inadequate infrastructure and living facilities making it difficult to form a reasonable town system. The inability to achieve agglomeration and scale effect seriously restricts residents’ low-carbon travel and development of villages and towns. With the overall shrinkage of towns due to population loss in recent years, the above problems are especially prominent [4,5]. To achieve regional carbon emission reduction targets in Northeast China, high-quality development of counties and coordinated urban–rural development need to be promoted. Implementing carbon emission reduction policies with clearer and more detailed objectives is necessary. This requires an in-depth study of the evolutionary characteristics of carbon emissions in Northeast China and an analysis of the differences in the spatial structure. Previous studies on the evolutionary characteristics of carbon emissions are mostly based on energy balance sheets provided by statistical yearbooks to calculate carbon emissions from energy consumption. Still, county-level statistics are limited to the total statistical energy consumption of industries above the scale, which cannot be accurately calculated by separating energy species in detail. Due to the lack of statistical data, the obtained data do not have spatial and temporal continuity. The above problems bring greater difficulties in calculating counties’ carbon emissions [6,7].

The Visible Infrared Imaging Radiometer Suite (VIIRS) sensor has gradually become an essential tool for monitoring the spatial and temporal evolution of spatial information and for analyzing national economic development [8,9,10]; it is carried by the National Polar-Orbiting Partnership (NPP), a US environmental monitoring satellite. It can detect human nighttime activity lights in the wavelength range of 0.5 to 0.9 μm, including urban nighttime lights and low-intensity nighttime lights generated by residential locations, traffic, etc. [11,12,13]. The types of nighttime lights it contains roughly correspond to the classification of production, building, and transportation sectors in the carbon emission inventory compilation, which can effectively reflect the carbon emissions generated by human activities. Many scholars in China and abroad have successfully applied nighttime light data to carbon emission estimation. Elvidge [14] et al. were the first to determine that they used time-series DMSP-OLS nighttime light images (day and night images obtained by the Operational Line scan System (OLS)of the Defense Meteorological Satellite Program (DMSP)) to identify greenhouse gas emissions associated with cities, towns, and industrial sites.

Many scholars have used the Intergovernmental Panel on Climate Change (IPCC) method to estimate carbon dioxide (CO_2_) emissions [15]. However, this method is based on administrative regions as statistical units, which limits the estimation of carbon emissions in prefecture level cities, county level cities, and smaller administrative divisions because statistical data are generally based on national or provincial units.

Oda et al. [16] proposed to use DMSP-OLS data to simulate carbon emissions in urban areas and population data to reflect carbon emissions in non-urban areas, resulting in a global carbon emission grid with a spatial resolution of 1 km. Raupach et al. [17] and Ghosh et al. [18] evaluated the spatiotemporal dynamics of carbon emissions from global energy consumption. In addition, Wang et al. [19] inverted the spatial status of China’s urban carbon emissions in 2013. At the same time, Meng [6] et al. also used a similar method to analyze the temporal and spatial variation characteristics of carbon emissions in urban areas of China. Relevant scholars have also conducted urban-scale carbon emission estimation studies. Lu [20] used DMSP-OLS data to estimate the distribution of carbon emissions in Hebei and Beijing based on human activity indices.

In terms of the factors influencing carbon emissions, relevant studies show that factors such as the living standard of residents, population size, economic level, urbanization level, and affluence are the main factors influencing the increase of regional carbon dioxide emissions and they show a positive correlation with carbon emissions [21,22,23,24,25,26]. The improvement of regional science and technology is, to a certain extent, conducive to reducing regional carbon emissions [27,28]. In addition, household carbon emissions also impact regional carbon emissions; raising residents’ low-carbon awareness can help reduce regional carbon emissions to a certain extent [29,30]. In terms of the level of influence of many factors on carbon emissions, Wang [31] et al. found in their study that the degree of influence on carbon emissions is population size, industrialization level, urbanization level, energy structure, technology level, and trade level in descending order. In addition, the compact urban form can reduce cities’ carbon emissions to some extent. On the contrary, loose urban form can lead to the growth of urban carbon emissions [32,33,34]. Most studies stay at the national and provincial levels, while there are fewer at the municipal and county levels.

Based on the SNPP-VIIRS (VIIRS is the Visible Infrared Imaging Radiometer Suite; SNPP is the Suomi National Polar-orbiting Partnership [35]) for calibration and integration data, this paper constructs a county carbon emission estimation model. It systematically analyzes the spatial and temporal evolution characteristics of carbon emissions in county towns and municipalities in Northeast China from 2012 to 2020. Based on this, the spatially hierarchical differentiation and the introduction of geographic detector technology reveal the factors influencing carbon emission spatial and temporal changes and the relationships between them. This study analyzes the relevant factors affecting carbon emissions in detail, considering various factors such as economic growth, industrial structure, population size, urbanization level, fiscal revenue and expenditure, and added value of secondary industry. The spatial-temporal evolution of carbon emissions in counties of Northeast China is based on GIS. It provides data support for low-carbon development and decision making in Northeast China. It provides a theoretical basis for counties in Northeast China to achieve “smart shrinkage” further and to guide the concentration of population and public resources in cities and towns.

## 2. Data Sources

### 2.1. Nighttime Lighting Data Source and Processing

This article’s 2012–2020 nighttime lighting data are from the SNPP/VIIRS nighttime lighting annual data (annual VNL V2). The spatial resolution is 15 arcsec and the image element radiation value is in nW/cm^2^/sr. The Earth Observation Group (EOG) EOG by NOAA/NGDC is based on the global VIIRS monthly cloud-free DNB composite, further filtered for extraneous features such as biomass burning, auroras, and background noise [13].

### 2.2. Nighttime Lighting Data Calculation

In general, the total light (total intensity) or average light (light density) intensity of an area can reflect the lighting characteristics of the area. This can be done by constructing the Sum of nighttime Lights (*SOL*), calculated in Equation (1)

(1) $$SOL=\overset{n}{\underset{i=1}{\sum }}DN_{i}$$

where *DN_i_* is the image element radiation value of each raster cell in the region; *n* is the number of raster cells in the region; *SOL* is the Sum of nighttime Lights in the region.

Although VIIRS/VNL/V2 (VNL2) has studied several issues dealing with satellite imaging system data technology and has significantly developed to improve the quality of nighttime light data information, the problem of gas combustion still needs to be solved. Gas flaring is the brightest source of radiation among all surface sources on Earth [8]. A large number of flares will be generated around it, which is undoubtedly a tremendously disturbing factor for constructing stable nightlight data. In this paper, we first cropped the VNL2 raster data of Northeast China to WGS84 vector maps using the geographic coordinate system, then converted them to the Asia Lambert planar projection coordinate system with the spatial resolution set to 500 m. By observing the nighttime light raster data of Northeast China for each year, we found a small number of negative values and individual extreme anomalies that gas-burning radiant light sources might cause. In this paper, we refer to the method of Elvidge [8] et al., to use 472.86 as the national cell image radiance extremes and to remove the cells with negative image values.

The study was conducted on a county-by-county basis. To make the study feasible, we combined the areas under the jurisdiction of each prefecture-level city into one whole. After dealing with missing data from several counties due to administrative division adjustments and missing data, one missing data from each of Heilongjiang, Jilin, and Liaoning. Namely, Qianguolos Mongolian Autonomous County, Karachi Zuoyi Mongolian Autonomous County, and Dulbert Mongolian Autonomous County. Finally, we counted the remaining 140 county research units. The county carbon emissions statistics do not include municipal districts where carbon emissions statistics are conducted.

**Figure 1:**
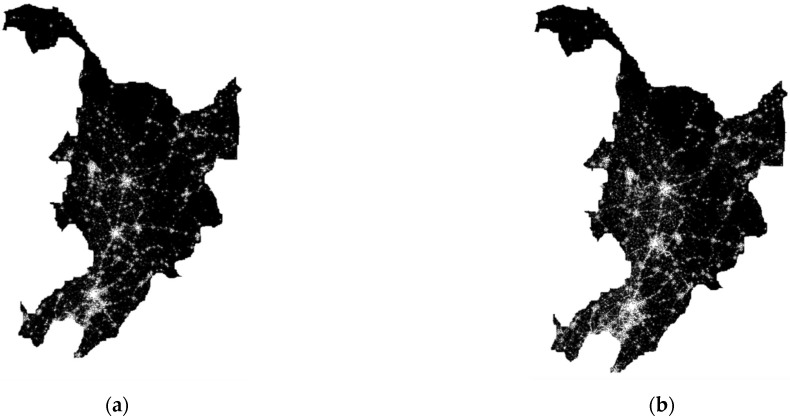
Nighttime lighting data in Northeast China in 2012 (**a**) and 2020 (**b**).

**Figure 2:**
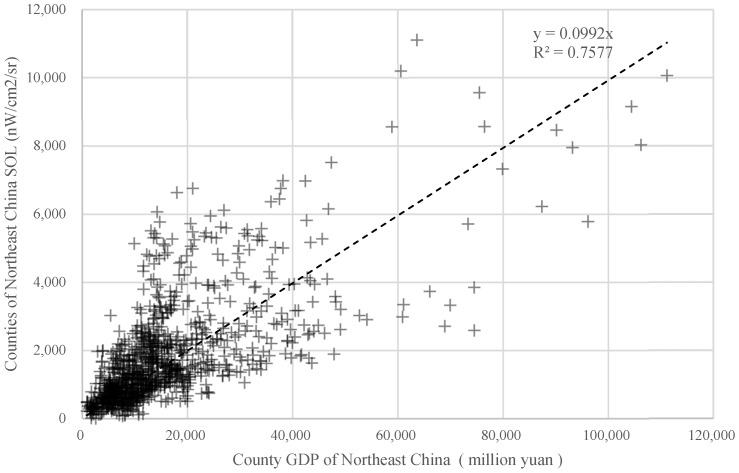
The fitting diagram of county GDP and county *SOL* in Northeast China from 2012 to 2020.

The processed VNL2 data were extracted from the county-level administrative boundary vector maps to obtain the total light image element radiation. The sum of nightlight radiation values and *SOL* values for 2012–2020 for 140 county-level administrative districts in Northeast China (Figure 1) matched with the Gross Domestic Product (GDP) data for 2012–2020 for 140 county-level administrative districts counted in the statistical yearbook. Correlation tests were performed, where the nominal GDP data for each county-level neighborhood were used for each province GDP deflator and converted to 2010 prices. The test results are shown in Figure 2. The coefficient of determination R^2^ of the fit between northeast county GDP and county *SOL* is 0.76. Based on the fact that the smaller the administrative range of the total light value extracted from satellite image data, the larger the error of the data, and the existence of a significant statistical error in county GDP itself, this paper considers that this coefficient of determination of 0.76 can already indicate a high fit between the two, suggesting that the county lighting data can better represent the economic development of each county-level administrative region.

### 2.3. Energy Statistics Sources

Energy consumption data for Northeast China were obtained from the energy balance sheets of each region in the *China Energy Statistical Yearbook* (2013–2020); the average low calorific value NCV of various energy sources was obtained from the *China Energy Statistical Yearbook 2017*; the CO_2_ emission factor CEF of different energy sources was obtained from the *2006 National Greenhouse Gas Emissions Inventory Guide*. The GDP of some counties and cities in Northeast China and the GDP index data of each province are from the *China Statistical Yearbook* (2013–2020).

## 3. Research Methodology

### 3.1. Calculation of Carbon Emissions from Energy Consumption

Based on the energy balance obtained from the *Chinese Energy Statistical Yearbook*, “Method 1” of IPCC (2006) and referring to Caiyi et al. [36], coal, coke, coke oven gas, blast furnace gas, converter gas, other gas, crude oil, gasoline, kerosene, diesel, fuel oil, natural gas, and LNG were selected from the energy balance. In total, 14 types of energy are chosen, including coal, coke, coke oven gas, blast furnace gas, converter gas, other gas, crude oil, gasoline, kerosene, diesel oil, fuel oil, liquefied petroleum gas, natural gas, and liquefied natural gas. Using these 14 types of energy consumption to calculate carbon dioxide emissions in Northeast China from 2012 to 2020, the specific estimation formula is shown in Equation (2).

(2) $$C=\overset{14}{\underset{i=1}{\sum }}C_{i}=\overset{14}{\underset{i=1}{\sum }}N_{i}\cdot NCV_{i}\cdot CEF_{i}$$

where *C* is the carbon dioxide emissions to be estimated; *i* denotes the 14 energy sources selected; *N_i_* represents the consumption of various energy sources; *NCV_i_* is the average low-level heat generation of different energy sources that is used to convert the consumption of various energy sources into energy units (TJ); *CEF_i_* denotes the carbon dioxide emission factor of various energy sources. The specific parameter values are shown in Table 1.

### 3.2. Carbon Emission Estimation Model Hypothesis

The previous section, according to the correlation test between the county lighting data and the GDP, shows that lighting data analyses can better reflect the continuous development of a country’s regional economy. Assuming that there is a linear correlation between carbon emissions and *SOL* values, the greater the *SOL* value, the more carbon there is. By constructing the linear equation of local carbon emission and lighting data, when considering the accuracy problem of reducing the scale to raster cells this paper refers to Du et al. [37,38,39]; it adopts the linear regression equation without intercept. The expression is:

(3) C=a×SOL

where *C* is the estimated carbon emissions; *SOL* is the total value of nighttime lighting data; *a* is the regression parameter.

### 3.3. Index Selection

This paper takes the value added of secondary industry, industrial structure, public finance income, public finance expenditure, and the urbanization rate as independent variables and regional carbon emissions as the dependent variable. This paper uses geographical detectors to explore the impact of various factors on carbon emissions. The main factors consist of five dimensions and seven indicators, as shown in Table 2.

The factors are Population (POP), GDP per capita (GDPP), the secondary industry as a share of GDP (SP), secondary industry added value (SE), financial revenue (INC) and financial expenditure (EX), and Urbanization Rate (UR). Due to the need for urban population statistics in Northeast China, the calculation of the urbanization rate is complicated. Therefore, the nighttime light index is chosen as an alternative indicator. The county-level unit data sets for 2012, 2016, and 2020 were constructed.

### 3.4. Geographic Detector

First proposed by the Institute of Geographical Sciences and Resources, Chinese Academy of Sciences, the advantage of geographic probes is that they can detect single-factor or two-factor interactions on the dependent variable. The exchange is generally identified by adding the product term of the two factors to the regression model and testing its statistical significance. The geographic detector q-statistic, which can be used to measure spatial heterogeneity, detect explanatory factors, and analyze interactions between variables, has been applied in multiple natural and social sciences fields. By calculating and comparing the *q*-values of every single aspect and the *q*-values of the superposition of two elements, the geographic detector can determine whether there is an interaction between two factors and the strength, direction, linearity, or nonlinearity of the interaction.

#### 3.4.1. Divergence and Factor Detection

Detects the spatial heterogeneity of *Y* (dependent variable) and how much of attribute *Y*’s spatial heterogeneity is explained by a specific factor *X* (independent variable). Using the *q*-value metric, the expression is:

(4) $$q=1-\frac{\sum_{h=1}^{L}N_{h}\sigma_{h}^{2}}{N\sigma^{2}}=1-\frac{SSW}{SST}$$

(5) $$SSW=\sum_{h=1}^{L}N_{h}\sigma_{h}^{2},\;SST=N\sigma^{2}$$

where *h* = 1, …, *L* is the stratification (strata) of variable *Y* or factor *X*, i.e., classification or partitioning; *Nh* and *N* are the number of cells in stratum *h* and the whole region, respectively; $\sigma_{h}^{2}$ and *σ*^2^ are the variances of *Y* values in stratum h and the entire region, respectively. SSW and SST are the sum of within-squares (Within the Sum of Squares) and the total variance of the whole area (Total Sum of Squares), respectively. The value of *q* is in the range [0, 1], and the larger the matter, the more significant the spatial heterogeneity of *Y*. If the independent variable *X* generates the stratification, the larger the value of *q* indicates the more substantial the explanatory power of the independent variable *X* on the attribute *Y*, and the weaker indicates the opposite. In the extreme case, a *q* value of 1 indicates that factor *X* completely controls the spatial distribution of *Y*, and a *q* value of 0 indicates that factor X has no relationship with *Y*. A *q* value means that *X* explains 100 × *q*% of *Y*.

#### 3.4.2. Interaction Detection

Interactive detection can quantitatively characterize the relationship between the two influence factors on the dependent variable, such as the influence factors A and B, and form a new influence factor C by the spatial superposition of A and B. The attributes of C are determined by A and B together. By comparing the influence of the A and B impact factors and the influence of impact factor C, it can be judged whether the influence of the interaction of the two factors on the dependent variable and the influence of the single factor on the dependent variable are stronger or weaker.

The interaction detection mainly has the following expressions: if P (A ∩ B) < min P (A), P (B), it indicates that the nonlinearity is weakened after the interaction of factors A and B; if min (P (A), P (B)) < P (A ∩ B) max (P (A), P (B)) and (A ∩ B) P (A) + P (B), the nonlinearity is strengthened after the interaction of A and B; if P (A ∩ B) = P (A) + P (B), A and B are independent of each other. The results are calculated using the geographic detector.

## 4. Results

### 4.1. Carbon Emission Estimation Model and Accuracy Check

#### 4.1.1. Carbon Emission Estimation Model

**Figure 3:**
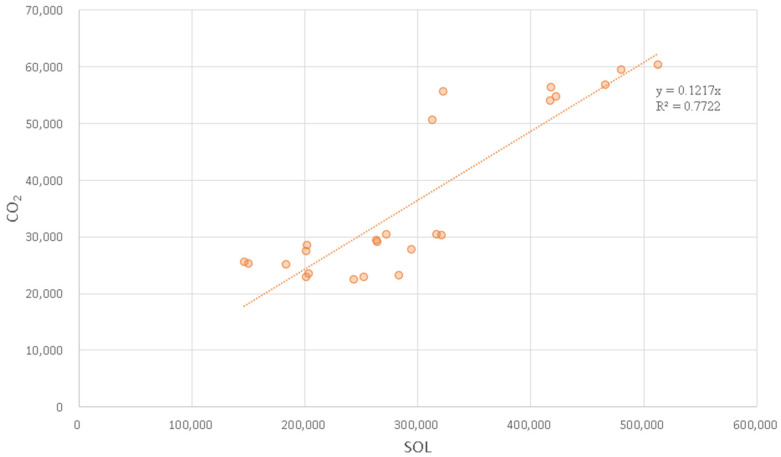
The fitting diagram of county CO_2_ and county *SOL* in Northeast China from 2012 to 2020.

Using nighttime lighting data and carbon emission statistics to construct a carbon emission estimation model, the estimation results are shown in Figure 3, which shows that the total value of *SOL* of nighttime lighting data and carbon emission statistics of energy consumption have a good linear correlation. The expression is:

(6) C=0.1217(0.0045)×SOL

The determination coefficient R^2^ of the regression equation is 0.7722, indicating that there is a high correlation between carbon dioxide and nighttime light data and the proportional coefficient is 0.1217. The estimated standard error is 0.0045, indicating that the proportional coefficient is significantly positive at the level of 1%, which is consistent with the expectation of this paper. Through the scale coefficient and the county light data, the carbon dioxide emission level of Northeast China can be estimated.

#### 4.1.2. Accuracy Check

To ensure the reliability of the carbon emission estimation model, the accuracy of the estimation results is further tested. Comparing the estimated results with the statistical carbon emissions, it can be found that the average relative error is 13.51%. See Table 3 for relative errors for each province and region for 2012–2020. On average, more than 80% of the provinces have an error value of less than 25%. They meet the estimation accuracy requirements and can provide a reliable database for the spatial and temporal evolution characteristics of carbon emissions in Northeast China.

### 4.2. General Characteristics of County Carbon Emissions in Northeast China

**Figure 4:**
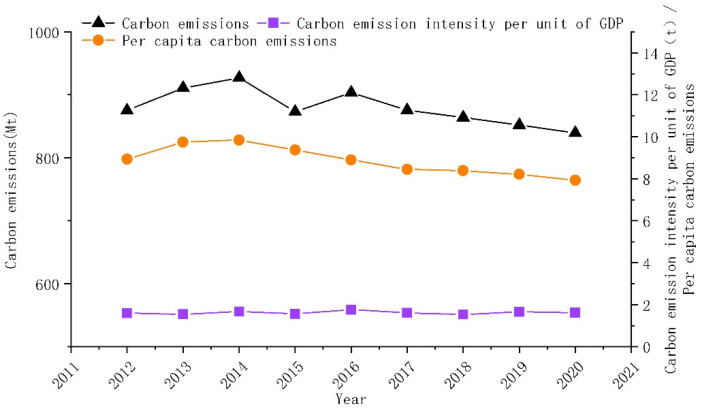
Changes in overall carbon emissions, per capita carbon emissions, and carbon intensity in Northeast China 2012–2020.

**Figure 5:**
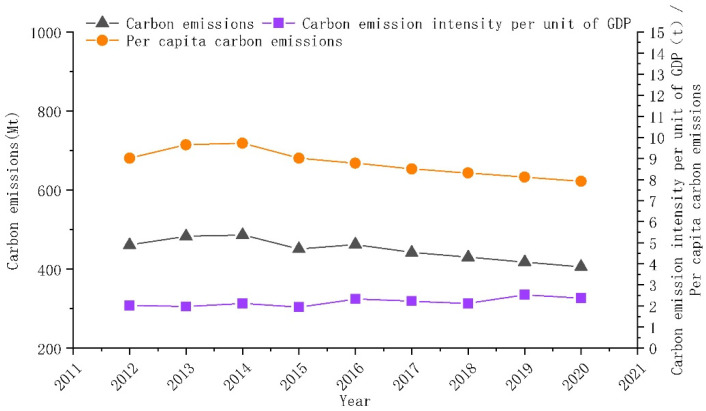
Changes in county-level carbon emissions, per capita carbon emissions, and carbon intensity in Northeast China 2012–2020.

Figure 4 and Figure 5 show the changes in carbon emissions, carbon intensity, and per capita carbon emissions in the whole (statistics include municipal districts) and the counties (statistics do not include municipal districts) of Northeast China. In Figure 4, the total carbon emissions in Northeast China grew and then slowly declined from 875.291 million tons in 2012 to 839.622 million tons in 2020. The average annual decline rate is 0.51%. In Figure 5, carbon emissions in the counties of Northeast China showed the same trend, with an upward change from 2012 to 2014 and a flat decline from 2014 to 2020. It decreased from 461.159 million tons in 2012 to 405.752 million tons in 2020, with an average annual decline rate of 1.5%. The carbon emissions in the counties of Northeast China experienced a two-stage evolution from 2012 to 2020: (1) from 2012 to 2014, carbon emissions rose, reaching a maximum of 486.325 million tons in 2014; (2) from 2014 to 2020, carbon emissions began to decline slowly, from 486.325 million tons in 2014 to 405.752 million tons in 2020. The average annual decline rate was 2.76%.

In Figure 5, the carbon emission per capita in the counties of Northeast China increased from 9.01 tons per capita in 2012 to 9.72 tons per capita in 2014 and decreased to 7.91 tons per capita in 2020. The average annual decline rate was 1.53%. Carbon emission intensity showed fluctuating changes from 2012 to 2020, increasing from 2.02 tons per capita in 2012 to 2.36 tons per capita in 2020. The average annual growth rate was 2.11%. The changes in per capita carbon emissions and carbon emissions showed different trends. The main reason was the gradual increase in population loss in Northeast China since 2014. Population loss leads to a decline in regional vitality, which promotes carbon emission reduction and per capita carbon emission increase.

### 4.3. Trends in Carbon Emissions by Region

Due to the different stages of economic development in each region, the county carbon emissions, per capita county carbon emissions, and county carbon emission intensity in each region showed specific stage differences.

Table 4 shows the county carbon dioxide emissions in Northeast China. From 2012 to 2020, the most significant county carbon dioxide emissions were in Liaoning Province. In 2012, the county carbon emissions in Jilin Province were more significant than those in Heilongjiang Province. From 2013 to 2017, the county carbon emissions in Heilongjiang Province were more significant than those in Jilin Province. From 2017 to 2020, there were subtle differences in county-level carbon emissions between Jilin and Heilongjiang.

Regarding per capita CO_2_ emissions in Northeast China counties from 2012 to 2020, Liaoning Province had the largest per capita CO_2_ emissions in the counties. The trend was slowly decreasing from 11.0 tons per capita in 2012 to 9.49 tons per capita in 2020. The average annual decline rate was 1.71%. CO_2_ emissions per capita in Heilongjiang Province showed a change of rising to a peak and then slowly decreasing. It grew from 11.01 tons per capita in 2012 to a maximum of 9.72 tons per capita in 2014. Then, it declined to 7.91 tons per capita in 2020. The county per capita CO_2_ in Jilin Province showed three stages: decreasing, increasing, and then slowly decreasing. It reduced from 8.65 tons per capita in 2012 to the lowest value, of 7.81 tons per capita in 2015, then rose to 8.52 tons per capita in 2017 and gradually and slowly decreased to 8.06 tons per capita in 2020.

The county carbon emission intensity in Liaoning Province increased from 2.1 tons per CNY 10,000 in 2012 to a maximum of 3.09 tons per CNY 10,000 in 2016. It gradually decreased to 2.71 tons per CNY 10,000 from 2016 to 2020. From 2012 to 2020, the average annual growth rate was 3.61%. The carbon emission intensity of Jilin Province counties decreased from 2.08 tons per CNY 10,000 in 2012 to 1.68 tons per CNY 10,000 in 2015, reaching a minimum value. It reached a maximum value of 2.72 tons per CNY 10,000 in 2019. The average annual growth rate was 2.76% from 2012 to 2020. The changing trend of carbon emission intensity in Heilongjiang Province counties was flat, reaching a maximum value of 2.15 tons per CNY 10,000 in 2014 and a minimum value of 1.75 tons per CNY 10,000 in 2018. Respectively, carbon emission intensity changed from 1.87 tons per CNY 10,000 to 1.82 tons per CNY 10,000 from 2012 to 2020. The average annual decline rate was 0.29%.

### 4.4. Trends in Carbon Emissions of Counties and Cities in Northeast China

**Figure 6:**
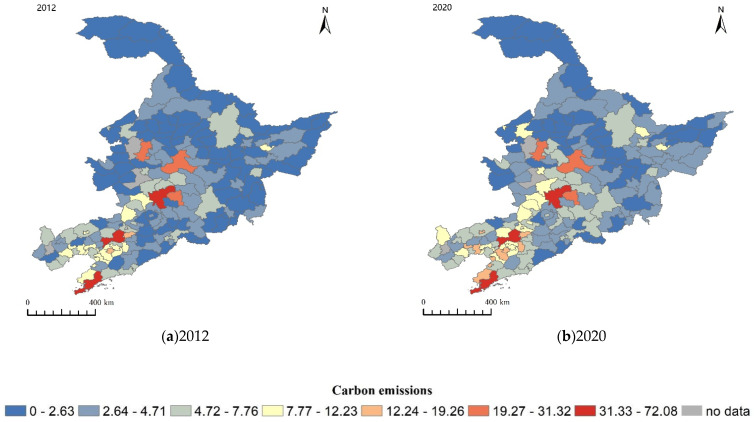
Spatial and temporal distribution of total carbon emissions in Northeast China in 2012 (**a**) and 2020 (**b**).

Figure 6 shows the spatial and temporal distribution of total carbon emissions in Northeast China in 2012 and 2020. In 2012, the high-value and medium-high-value areas of county carbon emissions were mainly concentrated near the municipal districts of the three provincial capitals and the southern coastal regions, while the medium-low- and low-value areas accounted for most of the Heilongjiang and Jilin provinces. In 2020, the high-value and medium-high-value regions of county carbon emissions showed a trend of gathering along the capital cities of the three provinces. The medium-value areas of county carbon emissions began to occupy most of Jilin Province and Liaoning Province. Most low and medium carbon emission areas still exist in Heilongjiang Province. The reason may be the gap between regional economic and technical levels, the degree of population loss gap, and different industrial structures.

**Figure 7:**
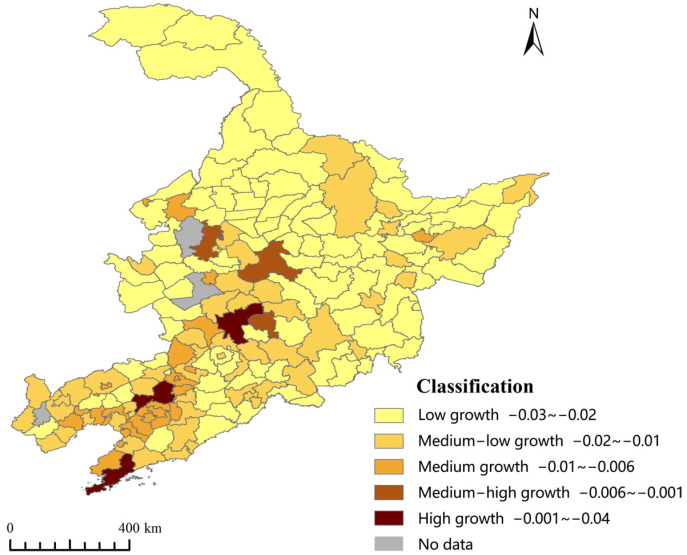
Classification of the rate of incremental carbon emissions in the counties of Northeast China 2012–2020.

In this paper, a breaking point model is used to classify the regional differences of carbon emissions based on GIS. It classifies the trends of total carbon emission growth of counties and cities from 2012 to 2020 into five types. These five types include low development, medium-low growth, medium growth, medium-high growth, and high growth, as shown in Figure 7. High-growth areas are concentrated in provincial capitals and first-tier cities. Counties and cities with medium-high growth rates are mainly distributed in the northern and coastal regions of Northeast China. They are characterized by gathering within counties and cities with high growth rates. The counties and cities with medium-low and low growth rates are mainly distributed in the southern part of Northeast China and some underdeveloped areas in the north.

**Figure 8:**
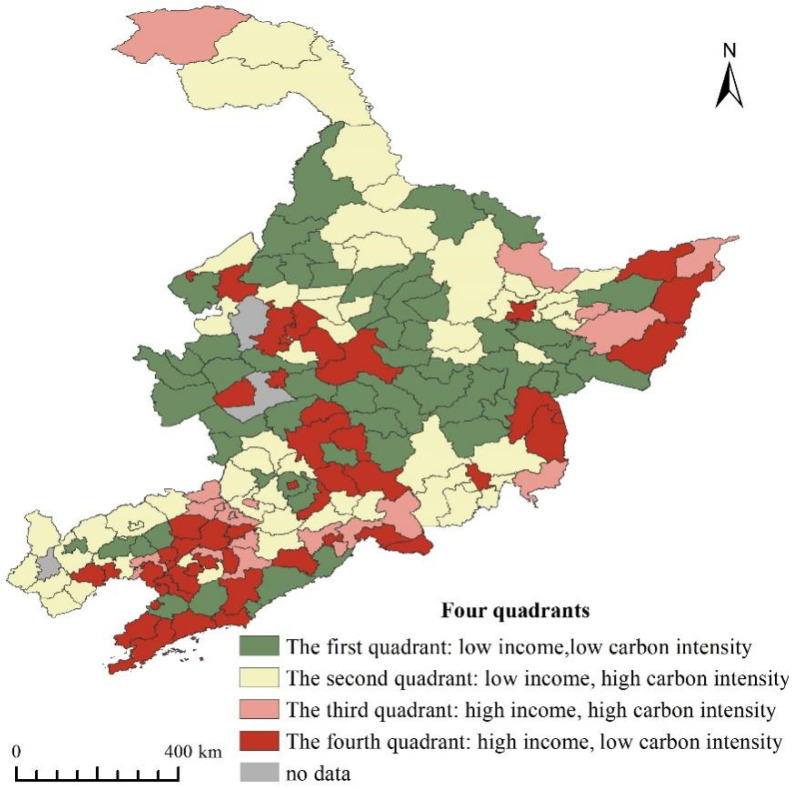
Four quadrants of GDP per capita and carbon intensity in Northeast China.

For regional carbon emission intensity and per capita income, the average emission level of the counties in Northeast China is used as the origin of the two-dimensional axis (i.e., the carbon emission intensity per unit of GDP is 2.18 tons per CNY 10,000 and the per capita GDP is CNY 41,200), which is divided into four quadrants, with the first quadrant characterized by low income and low carbon emission intensity. In contrast, the second quadrant is characterized by low income and high carbon emission intensity. The third quadrant is characterized by high income and high carbon emission intensity. In contrast, the fourth quadrant is characterized by high income and low carbon emission intensity. This distribution map divides all counties and cities in Northeast China into corresponding quadrants and the resulting distribution map is shown in Figure 8, where we can observe the following four characteristics.

The fourth quadrant is characterized by high income and low carbon emission intensity. It is mainly located near the municipal districts of the capital cities of Northeast China and the southern coastal areas. These are the earliest industrialized areas. These regions may play a leading role in reducing carbon emissions in Northeast China, promoting the transformation of old and new kinetic energy, and promoting industrial optimization and upgrading.

The third quadrant is characterized by relatively high income and high carbon emission intensity. It is mainly located in the southern areas of Northeast China. These areas are now in the stage of rapid industrialization. The existing heavy chemical industry is recommended to be upgraded through technology to improve energy efficiency in order to make it a “green industry”.

The second quadrant is characterized by relatively low income but high carbon emission intensity. It is mainly distributed in Liaoning and Jilin provinces, with linear characteristics. The rest of the quadrant is distributed in the northern part of Heilongjiang Province. They are currently in the early stages of industrialization and the industrial base is relatively weak. In the process of development, it is recommended to optimize the energy structure and improve economic efficiency to adapt to the development stage.

The first quadrant is characterized by low income and low carbon emission intensity, with a linear distribution in the central part of Northeast China that gradually extends to the north. It is suggested to speed up the construction of a modern agricultural system with regional characteristics with obvious advantages and outstanding benefits, promote steady economic growth, and promote the continuous increase of residents’ income.

### 4.5. Analysis of Influencing Factors

#### 4.5.1. Detection of Single Factor

As shown in Table 5, overall, in 2012, the detector *q* values are as follows: SE (0.568) > INC (0.56) > EX (0.548) > POP (0.421) > UR (0.413) > SP (0.220) > GDPP (0.153). In 2020, the detector *q* values are as follows: UR (0.648) > INC (0.551) > SE (0.494) > POP (0.409) > EX (0.26) > SP (0.195) > GDPP (0.03). It can be seen that the greatest influence on the spatial heterogeneity of carbon emissions shifted from SE in 2012 to UR in 2020. Affluence did not have a significant effect on the growth of carbon emissions.

Population size had some explanatory power on the spatial divergence of carbon emissions and showed a decreasing trend. *q* values of POP in 2012 and 2016 were 0.421 and 0.41, respectively, indicating that POP was the most explanatory factor in 2012 and 2016. Subsequently, the value decreased to 0.409 in 2020, indicating that the explanatory power of POP on the spatial divergence of carbon emissions decreased.

The explanatory power of GDP per capita (GDPP) for the spatial divergence of carbon emissions showed a trend of increasing before decreasing, with the *q*-value increasing from 0.153 in 2012 to 0.305 in 2016 and finally reducing to 0.3 in 2020 and showing no correlation. The increase in GDP per capita between 2016 and 2020 did not significantly lead to the rise in carbon emissions of residents, probably due to the population continued loss of people and a decrease in urban dynamics.

Overall, the spatial divergence of carbon emissions is explained by the value added of secondary production (SE) relative to the share of value added of the secondary output in GDP (SP). Both show a decreasing and increasing trend, but the value added of secondary production as a share of GDP (SP) decreased to 0.1 in 2016, showing no correlation. *q* values for value-added secondary display in 2012 and 2020 were 0.568 and 0.494, respectively, much more significant than 0.22 and 0.195 for the value-added of the secondary output as a share of GDP (SP).

The influence of INC on carbon emissions was consistently more significant than that of EX, which was relatively weak in explaining the spatial variation of carbon emissions. The *q*-values for the three years of the study were 0.548, 0.288, and 0.26, respectively, with relatively low overall values and a decreasing trend.

The explanatory power of UR on the spatial divergence of carbon emissions showed a rise before a fall. The *q*-values of the three years were 0.413, 0.714, and 0.648, respectively, and although it decreased to 0.648 in 2020, it maintained a high level of influence in general. At this stage, the vast rural areas in Northeast China are more dependent on the primary industry. They do not produce significant material carbon emissions, that are mainly generated by the counties.

#### 4.5.2. Detection Interactions

Table 6 summarizes the top 10 interaction factors in 2012, 2016, and 2020. The results show a significant increase in the interactions between the factors. Four observations can be drawn as follows: (1) Urbanization Rate (UR) and public revenue (INC), which have high *q*-values when interacting with each other, indicate that affluence and urbanization levels play a decisive role in the impact of carbon emissions in the region. For example, in 2012, 2016, and 2020, the *q*-values when UR and INC interacted reached 0.87, 0.80, and 0.79, respectively, and maintained a high level of influence despite the decreasing trend. (2) When the factors reflecting the economic level and population size interacted, the *q*-values showed a decreasing trend and maintained a relatively low level of influence. For example, the *q*-values for the three years in the study were 0.733, 0.69, and 0.666, respectively. (3) Other factors had high *q*-values when they interacted with UR. For example, the *q*-values of INC, SE, and SP interacting with UR in 2012 reached 0.87, 0.789, and 0.773, respectively. In 2016 and 2020, the top 10 interacting influences had as many as six interacting influences with UR. (4) The degree of influence on carbon emission when the two factors of population size and industrial structure interacted was maintained at a low level; it decreased from 0.701 in 2012 to 0.687 in 2020. 

## 5. Policy Recommendations

(1) Through the study, it is found that the overall carbon emissions of counties in Northeast China reached a peak by 2014. Still, there are significant differences in their carbon emissions due to the different factors such as industrial structure, energy structure, and economic level in other regions. Since 2012, the phenomenon of population loss in Northeast China provinces has intensified rapidly and the phenomenon of urban contraction and county contraction has become more serious, especially in counties and towns. The loss of population has led to a decline in regional vitality, but it is conducive to the reduction of carbon emissions. Therefore, in the context of carbon peak, we should strictly control the scale of development, activate the stock, and improve the efficiency of resource allocation.

(2) By studying the carbon emission influencing factors in counties in this paper, it is found that urbanization rate and industrial structure have a greater degree of influence on regional carbon emission. Urbanization means the formation of a dynamic and competitive regional economy, implies the integration of regional resources, and has an essential impact on regional economic development. Therefore, in the urbanization process, the Northeast China provinces should optimize the layout, structure, function, and development mode of infrastructure. The agglomeration effect is formed. We must strive to avoid the growth of carbon emissions in the future urbanization rate. At the same time, we must also promote industrial restructuring. We should improve the efficiency of the secondary industry while actively developing the tertiary industry and stabilizing the primary industry.

(3) By analyzing the spatial and temporal distribution of county carbon emissions in Northeast China from 2012 to 2020, this paper finds that high-carbon counties in Northeast China were mainly concentrated in the surrounding areas of provincial capitals and coastal areas in Northeast China from 2012 to 2020. Therefore, we should accelerate the promotion of industrial agglomeration, enterprise concentration, and resource-intensive development. The resources of counties are close to cities and the spatial layout of the region should be adjusted in a planned manner, which includes concentrated construction land, infrastructure, etc. The waste of resources due to population loss and a series of problems such as low vitality, low efficiency, and low living standards arising from the small size and scattered distribution of counties must be avoided.

## 6. Conclusions

The conclusions of this paper are as follows:

(1) The accuracy of the county-level carbon emission inversion model in Northeast China is relatively high. The determination coefficient R^2^ of the regression equation is 0.7722. It indicates that there is a high correlation between carbon dioxide and nighttime light data. The proportional coefficient is 0.1217. More than 80% of the provinces have an error of less than 25%, meeting the estimation accuracy requirements. It indicates that nighttime lighting can explain the carbon emission data of counties in Northeast China.
(2) From 2012 to 2020, carbon emissions in county towns in Northeast China showed a trend of rising before falling, increasing from 461.159 million tons in 2012 to 486.325 million tons in 2014 and slowly falling to 405.752 million tons in 2020. Per capita carbon emissions show the same trend, increasing from 9.01 tons per capita in 2012 to 9.72 tons per capita in 2014. It then decreased to 7.91 tons per capita in 2020. In conclusion, the carbon emissions in the counties of Northeast China showed a convergence trend and reached a peak in 2014.
(3) High growth areas of carbon emissions are concentrated in provincial capitals and first-tier cities. The counties with medium-high growth rate are mainly distributed in the northern and coastal areas of Northeast China. These areas are characterized by concentrated distribution around provincial capitals. The counties and towns with medium-low and low growth rates are mainly distributed in the underdeveloped areas in the north and south in Northeast China.
(4) This analysis analyzes the degree of single and interactive influences of economic level, population size, urbanization rate, industrial structure, and public finance revenue and expenditure on carbon emissions in the counties of Northeast China using the geographic detector method. The results show that, under the single influence factor, the most influential factor on county carbon emissions in 2012 was the value added of secondary production. The most influential factor in 2016 and 2020 was the urbanization rate. Under the two-factor interaction, it is found by comparison that other factors showed a higher level of influence on county carbon emissions when interacting with the urbanization rate.

## Figures and Tables

**Table 1:** NCV and CEF parameter values.

Energy Name	Coal Coke	Coke	Coke Oven Gas	Blast Furnace Gas	Converter Gas	Other Gas	Crude Oil
NCV (kj/kg)	20,908	28,435	17,981	3855	8585	18,273.6	41,816
CEF (kg/TJ)	95,977	105,996	44,367	259,600	181,867	44,367	73,333
**Energy Name**	**Gasoline**	**Kerosene**	**Diesel**	**Fuel Oil**	**Liquefied Petroleum Gas**	**Natural Gas**	**Liquefied Natural Gas**
NCV (kj/kg)	43,070	43,070	42,652	41,816	50,179	38,931	44,200
CEF (kg/TJ)	70,033	71,500	74,067	77,367	63,067	56,100	64,167

Sources: (1) NCV values from *China Energy Statistical Yearbook 2017*; LNG values from IPCC (2006). (2) CEF values from IPCC (2006).

**Table 2:** Influencing factors.

Variable Type	Variable Name	Variable Meaning
Population	POP	Total population at the end of the year (10,000 people)
GDP per capita	GDPP	Per capita GDP (CNY)
Industrial structure	SE	Secondary industry added value
	SP	Secondary industry added value/GDP
Financial revenue and expenditure	INC	Local fiscal revenue (CNY 10,000)
	EX	Local fiscal expenditure (CNY 10,000)
Urbanization rate	UR	County resident population/total population

**Table 3:** Relative error of carbon emission estimation model.

Region	2012	2013	2014	2015	2016	2017	2018	2019	2020
Liaoning Province	25%	29%	10%	6%	6%	1%	2%	3%	4%
Jilin Province	30%	28%	11%	5%	7%	24%	22%	19%	21%
Heilongjiang Province	14%	11%	11%	9%	9%	23%	25%	22%	23%
Northeast China	23%	24%	5%	1%	1%	14%	13%	20%	21%

**Table 4:** Natural emissions, carbon emissions per capita, and carbon emission intensity by province over time 2012–2020.

	Carbon Emissions (Million Tons)			Carbon Emissions Per Capita (Ton/Per Capita)			Carbon Emission Intensity (Ton/Per CNY Ten Thousand)		
	Heilongjiang	Jilin	Liaoning	Heilongjiang	Jilin	Liaoning	Heilongjiang	Jilin	Liaoning
2012	112.750	131.543	216.867	7.393	8.650	11.002	1.872	2.087	2.110
2013	142.141	128.372	212.410	9.489	8.505	10.945	2.112	1.878	1.936
2014	143.099	130.219	213.008	9.564	8.675	10.930	2.154	1.954	2.240
2015	136.146	118.016	196.805	9.059	7.877	10.110	1.972	1.688	2.166
2016	141.537	120.532	200.112	7.734	8.190	10.398	2.046	1.836	3.096
2017	121.766	123.023	197.322	6.729	8.526	10.246	1.760	1.874	3.053
2018	120.985	118.180	190.874	6.745	8.254	9.928	1.757	1.865	2.732
2019	115.124	116.273	186.499	6.458	8.187	9.709	2.009	2.725	2.866
2020	109.263	114.366	182.123	6.175	8.065	9.496	1.828	2.547	2.719

**Table 5:** Detection results for influencing factors.

Index	*q*		
	2012	2016	2020
POP	0.421 ***	0.410 ***	0.409 ***
GDPP	0.153 ***	0.305 ***	0.030
SE	0.568 ***	0.259 ***	0.494 ***
SP	0.220 ***	0.10	0.195 ***
INC	0.560 ***	0.374 ***	0.551 ***
EX	0.548 ***	0.288 ***	0.260 ***
UR	0.413 ***	0.714 ***	0.648 ***

Note: *** *p* < 0.01.

**Table 6:** Detection results of interaction for influencing factors.

Interacting Factors	2012	Interacting Factors	2016	Interacting Factors	2020
UR ∩ INC	0.870	UR ∩ GDPP	0.812	UR ∩ INC	0.799
UR ∩ SE	0.789	UR ∩ EX	0.804	UR ∩ SE	0.796
UR ∩ SP	0.773	UR ∩ INC	0.803	UR ∩ GDPP	0.796
POP ∩ INC	0.760	UR ∩ SP	0.782	UR ∩ SP	0.792
POP ∩ GDPP	0.733	UR ∩ SE	0.775	UR ∩ EX	0.788
POP ∩ SE	0.723	POP ∩ UR	0.761	POP ∩ SE	0.735
UR ∩ EX	0.710	GDPP ∩ SE	0.711	UR ∩ POP	0.720
POP ∩ SP	0.701	INC ∩ GDPP	0.710	POP ∩ INC	0.708
EX ∩ INC	0.696	EX ∩ GDPP	0.703	POP ∩ SP	0.687
SE ∩ EX	0.692	POP ∩ GDPP	0.690	POP ∩ GDPP	0.666

## Data Availability

Not applicable.
